# What Can You See? Identifying Cues on Internal States From the Movements of Natural Social Interactions

**DOI:** 10.3389/frobt.2019.00049

**Published:** 2019-06-26

**Authors:** Madeleine E. Bartlett, Charlotte E. R. Edmunds, Tony Belpaeme, Serge Thill, Séverin Lemaignan

**Affiliations:** ^1^Centre for Robotics and Neural Systems (CRNS), University of Plymouth, Plymouth, United Kingdom; ^2^Warwick Business School, University of Warwick, Coventry, United Kingdom; ^3^ID Lab—imec, University of Ghent, Ghent, Belgium; ^4^Interaction Lab, School of Informatics, University of Skövde, Skövde, Sweden; ^5^Donders Institute for Brain, Cognition, and Behavior, Radboud University, Nijmegen, Netherlands; ^6^Bristol Robotics Lab, University of the West of England, Bristol, United Kingdom

**Keywords:** social psychology, human-robot interaction, machine learning, social interaction, recognition

## Abstract

In recent years, the field of Human-Robot Interaction (HRI) has seen an increasing demand for technologies that can recognize and adapt to human behaviors and internal states (e.g., emotions and intentions). Psychological research suggests that human movements are important for inferring internal states. There is, however, a need to better understand what kind of information can be extracted from movement data, particularly in unconstrained, natural interactions. The present study examines which internal states and social constructs humans identify from movement in naturalistic social interactions. Participants either viewed clips of the full scene or processed versions of it displaying 2D positional data. Then, they were asked to fill out questionnaires assessing their social perception of the viewed material. We analyzed whether the full scene clips were more informative than the 2D positional data clips. First, we calculated the inter-rater agreement between participants in both conditions. Then, we employed machine learning classifiers to predict the internal states of the individuals in the videos based on the ratings obtained. Although we found a higher inter-rater agreement for full scenes compared to positional data, the level of agreement in the latter case was still above chance, thus demonstrating that the internal states and social constructs under study were identifiable in both conditions. A factor analysis run on participants' responses showed that participants identified the constructs *interaction imbalance, interaction valence* and *engagement* regardless of video condition. The machine learning classifiers achieved a similar performance in both conditions, again supporting the idea that movement alone carries relevant information. Overall, our results suggest it is reasonable to expect a machine learning algorithm, and consequently a robot, to successfully decode and classify a range of internal states and social constructs using low-dimensional data (such as the movements and poses of observed individuals) as input.

## 1. Introduction

One of the main goals in the field of Human-Robot Interaction (HRI) is to create robots capable of recognizing and adapting to human interaction partners in an appropriate manner (Dautenhahn and Saunders, 2011). In human-human interactions, the appropriateness of our responses to others is often a result of our ability to recognize the internal states (e.g., intentions, dispositions) of our interaction partner (Domes et al., 2007). Here we focus on internal states and social constructs relevant to task engagement and social relations between interaction partners. For example, we consider states that can be thought of as dispositional judgments (e.g., friendliness), states which can be considered emotional and are embedded within a social context (e.g., aggression), and states relevant to task performance (e.g., boredom). These states are communicated through both verbal and non-verbal cues (Pollick et al., 2001; Manera et al., 2011). Endowing robots and behavior classification systems with a similar ability to recognize internal states based on non-verbal behaviors would allow for more appropriate, autonomous human-robot interactions (Breazeal et al., 2009; Vernon et al., 2016), and for classification systems to provide more detailed insights into human behavior, e.g., for security purposes (Gowsikhaa et al., 2014).

### 1.1. Internal State Recognition

HRI research exploring approaches to achieving on-line recognition of human internal states/behavior draws on our understanding of how humans themselves infer internal states and social constructs. For example, a rich history of research has led to the assumption that humans are able to infer the internal states of others by observing their actions and movements (Gallese and Goldman, 1998; Manera et al., 2011; Quesque et al., 2013) and facial expressions (Ekman and Friesen, 1971; Haidt and Keltner, 1999; Tracy and Robins, 2008). In their paper, Manera et al. (2011) claim that “*in some circumstances, the movement of a human body…is sufficient to make judgments…in relation to the actor's intention"* [p. 548]. The idea here is that our intentions or emotions influence differences in the movements we make and, as observers, we are able to pick up on these differences and use them to infer the internal state of the person performing the action (Pollick et al., 2001; Ansuini et al., 2014; Becchio et al., 2017). To examine this researchers have used point-light displays and other methods to isolate movement information from other sources of information. Point-light displays denote the position and movements of an actor's joints on an otherwise blank display. Studies using this type of stimulus have shown that humans are able to use observed movement to infer an actor's gender (Kozlowski and Cutting, 1977; Mather and Murdoch, 1994; Hufschmidt et al., 2015), intention (Manera et al., 2010; Quesque et al., 2013) and emotional state (Pollick et al., 2001; Alaerts et al., 2011).

Available evidence also suggests that internal states and social constructs which fall under our definition of being socially relevant, dispositional or related to task engagement/performance are recognizable from observable movement. Okada et al. (2015) found that observable movements and non-verbal audio information produced during spontaneous, naturalistic interactions were sufficient for classifying dispositions and social behaviors such as dominance and leadership. Similarly, Sanghvi et al. (2011) demonstrated that postural behaviors could be used to classify a child's engagement with a robotic opponent, with which the children are playing a game. Beyan et al. (2016) asked four unacquainted individuals to complete a group decision task. They found that a classifier, when fed the 3D positional data of the interaction, was able to identify leaders within the group based on head pose and gaze direction information. Sanchez-Cortes et al. (2011) applied a computational framework to the inference of leadership and related concepts (e.g., dominance, competence) from non-verbal behaviors in a group interaction. Interactions in this study took place between four previously unacquainted individuals whose interactions were spontaneous and minimally structured. Sanchez-Cortes and colleagues were able to identify which behaviors were most informative for the recognition of the different leadership concepts. For example, conversational turn-taking and body movement behaviors were found to be the most informative for inferring leadership, whereas head activity and vocal pitch were the most informative for inferring competence.

States which are socially relevant, dispositional or task related, (such as friendliness, dominance or engagement) are particularly relevant for HRI research where the aim is to provide a socially interactive agent. In such scenarios it is preferable to have an agent which can provide appropriate social behaviors and responses (Dautenhahn and Saunders, 2011). Whilst emotion and intention recognition are definitely important for generating appropriate autonomous social behaviors from a robot, some HRI scenarios would also benefit from an ability to recognize internal states as we have defined them here. For instance, a teaching robot, such as those developed by the L2TOR project (Belpaeme et al., 2015), would be better able to provide appropriately timed encouragements or prompts if able to recognize when a student is bored or not engaged with the learning task.

As a result, HRI researchers have begun exploring ways in which observed movement can be utilized by robots and artificial systems to enable automated interpretation of, and responding to, the internal states of humans (Schrempf and Hanebeck, 2005; Han and Kim, 2010). Whilst humans also use other cues such as tone of voice (Walker-Andrews, 1997), findings such as those described above suggest that movement information may be sufficient for recognizing some, if not all, human internal states.

### 1.2. Current Study

#### 1.2.1. Motivation and Approach

To take advantage of this information for the purposes of internal state recognition it is important to first identify what internal state information is available in movements and body postures. This knowledge is particularly useful for streamlining the design process for a robot or classifier able to interpret such data. For example, if we want to design a system able to recognize when a human is bored, we first need to know what data is sufficient, if not optimal, for recognizing this state. Would the system need to take multiple behaviors into account, e.g., movements and prosodic features, or would movement alone be enough? In the case of internal states such as emotions and intentions, previous research suggests that movement information is sufficient for gaining insight (e.g., Tracy and Robins, 2008; Manera et al., 2011; Quesque et al., 2013). Given that the aim of HRI research is to create systems and robots which can be deployed in the real world, it is also important to consider that a classifier must be able to deal with natural, spontaneous human behaviors. Consequently, it is important to explore whether (and which) internal states can be recognized from the movements produced in natural human interactions. A a growing pool of studies have examined this (e.g., Sanchez-Cortes et al., 2011; Sanghvi et al., 2011; Shaker and Shaker, 2014; Okada et al., 2015; Beyan et al., 2016; Okur et al., 2017; Kawamura et al., 2019). However, further research is needed to provide a better understanding of which internal states can be inferred from such movements.

We therefore propose that an exploration into how readily different types of internal states can be identified from naturalistic human behavior would be beneficial for the streamlining of future HRI research. That is, by identifying which internal states are best recognized from a particular behavioral modality (e.g., biological motion), future research can identify which data sources are most useful for a given recognition task.

This study takes the first steps in this direction by developing a method for determining which internal state information is reported as identifiable by humans when they observe people in natural interactions. Given the strength of evidence suggesting that movement information is useful for identifying emotional and other internal states or social constructs (e.g., Pollick et al., 2001; Gross et al., 2012; Quesque et al., 2013; Beyan et al., 2016), this modality is likely to be a rich source of internal state information. Further, by extending this work to naturalistic interactions, we will find which internal states are likely to be identified in more ecologically valid settings. The usefulness of these states to HRI, indicate that an exploration of which internal states, from a selection of several, are recognizable from human movements would be helpful in guiding future research and development. To address this, we aim to examine and compare how reliably humans report identifying a number of different internal states and social constructs from observable movements.

To summarize, the main aim of this study is to demonstrate a method for identifying: (1) whether the data source of choice (in this case observable movements) can be used by humans to infer internal states and social constructs, and (2) what internal states and social constructs are readable from the movements within the data set. To do so, we will present short video clips of social interactions (exhibiting seven different internal states and social constructs) to participants. These clips come from the PInSoRo (Lemaignan et al., 2017) data set made openly available by our group^1^. This data set consists of videos of child-child or child-robot interactions. Children were asked to play for as long as they wanted on a touch-screen table-top device. For this study, we will solely use the child-child interactions as these are more likely to involve spontaneous behaviors throughout the children's interactions with one another. Some participants will view short clips including the full visual scene (full-scene condition) and others clips containing only movement and body posture information (movement-alone condition). These clips will contain at least one noticeable internal state (for details of the selection process see the Method section). Following each clip, participants respond to a series of questions where they can describe the internal states (e.g., boredom, friendliness) or social constructs (e.g., cooperation, dominance) they identified in the children's behaviors. By comparing responses in each condition we expect to be able to identify constructs which are likely to be recognizable from movement information alone.

#### 1.2.2. Hypotheses and Predictions

Based on previous findings that humans are able to recognize internal states such as emotions (Gross et al., 2012) and group dynamics such as leadership (Beyan et al., 2016) from human motion information, we expect the following:

1. Participants will report being able to draw internal state information from the movement-alone videos (Hypothesis 1). Specifically, we predict that even in the impoverished movement-alone condition, the provided ratings will be sufficient to describe the internal states and social constructs identified in the observed interaction. This can be tested by training a classifier on the full-scene ratings, and assessing its performance when tested on the movement-alone ratings.
2. However, given that participants in this condition are provided with fewer visual cues than those viewing the full-scene videos (e.g., lack of resolution for facial expressions) we expect a higher recognition error rate in the movement-alone condition compared to the full-scene condition (Hypothesis 2). If this is the case, we predict that inter-rater agreement levels amongst participants will be above chance in both conditions (i.e. the same constructs are robustly identified in the clips by the participants), but with higher levels of agreement in the full-scene condition.

## 2. Method

### 2.1. Design and Participants

**Figure 1:**
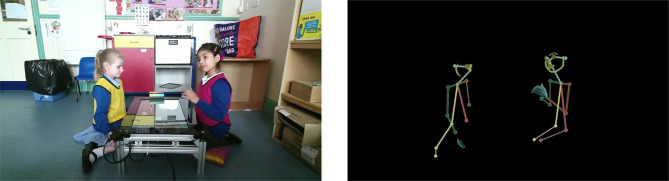
Captures of one of the twenty video-clips, *full-scene* condition on the left, *movement-alone* condition on the right. Written consent for these images to be shared was obtained during collection.

This study examined the effect of video type (full-scene vs. movement-alone) on responses to questions about the nature of the interaction depicted in the videos. We used a between-subject design: participants saw either *full-scene* clips (Figure 1, left) or *movement-alone* clips (Figure 1, right). 284 participants were recruited from Amazon's Mechanical Turk (MTurk). A total of 85 participants were excluded from analysis due to incorrect answers to an attention check (discussed in Procedure), leaving 199 participants (see Table 1 for demographics). All participants were remunerated $1 (USD) upon completion of the experiment.

**Table 1:** Demographics of participants included in the analyses.

**Condition**	***N***	**Mean Age (Range)**	**Gender (%M, %F)**	**% American**	**% English First Language**
Movement-Alone	100	34.52 (22–70)	55%, 44%	75%	80%
Full-Scene	99	33.54 (19–72)	65%, 34%	69%	73%
Both	199	34.03 (19–72)	60%, 39%	72%	76%

### 2.2. Materials

The stimuli used for this experiment were extracted from the PInSoRo data set. This data set contains videos (up to 40 min long) of pairs of children interacting whilst playing on a touch-screen table-top. For the present study we extracted twenty 30 s clips from these videos. We wanted to provide participants with clips which showed both children in the frame at the same time. We therefore selected our stimuli from videos filmed using a camera which had been positioned roughly 1.4m away from the touch-screen table-top, with the table-top in the center of the camera's view, thus allowing for each child to be viewed on either side of the frame (see Figure 1, left).

Two versions of the same clips were extracted: the *full-scene* clips were the raw video footage of the children playing, recorded from a static camera (Figure 1, left); the *movement-alone* clips were based on the exact same clips, but post-processed to extract skeletal and facial landmarks (using the OpenPose library^2^; Cao et al., 2017). Resulting landmarks were rendered on a black background, and connected to each other using colored lines, so that each child was depicted as a stick-man-style figure (Figure 1, right).

Clip selection was made based on whether a notable “event” or social dynamic occurred, defined as the labels listed in Table 2. This was done by watching the full-scene clips and working out what internal states and social constructs might be inferred from the children's movements. Specifically, two experimenters selected and labeled clips (by first independently extracting and annotating clips from the PInSoRo dataset, and second discussing to reach consensus) wherein at least one of the following seven concepts described the children's behavior or their interaction in the full-scene clips (see Table 2):

1. Boredom - at least one child was bored or not engaging with the task on the touch-screen (e.g., resting head in hand, interacting with touch-screen in slow/lazy manner).
2. Aggression - at least one child exhibited a physical aggressive action either toward the touch-screen or the other child (e.g., hitting the screen, pushing the other child's hand away).
3. Cooperation - the children were working together and/or communicating about how to perform a task [e.g., talking, joint attention (looking at the same object together), nodding].
4. Dominance - one child was bossy, performing most of the actions on the touch-screen or clearly in charge (e.g., pointing to touch-screen and talking at the other child, stopping the other child from using the touch-screen, being the only child to use the touch-screen).
5. Aimless play - at least one child was interacting with the touch-screen in a non-goal-directed manner or without being very engaged in their task (e.g., sitting slightly away from touch-screen whilst still using it, slow/lazy movements on touch-screen, not always looking at what they're doing).
6. Fun - at least one child was having fun (e.g., laughing, smiling).
7. Excitement - at least one child behaved excitedly (e.g., more dynamic than just “having fun," hearty laughter, open smiling mouth, fast movements).

**Table 2:** Labels that experimenters assigned to each clip during clip selection.

**Clip**	**Label 1**	**Label 2**	**Label 3**
01	Aggressive		
02	Aggressive	Excited	Aimless
03	Excited	Fun	
04	Cooperative		
05	Bored	Aimless	
06	Cooperative		
07	Dominance		
08	Bored		
09	Cooperative		
10	Cooperative	Dominance	
11	Cooperative	Dominance	
12	Aggressive	Aimless	
13	Excited	Aggressive	Aimless
14	Aggressive	Fun	
15	Dominance		
16	Cooperative	Dominance	
17	Excited	Aggressive	
18	Aggressive	Dominance	
19	Dominance		
20	Excited		

It was decided that multiple labels could be applied to each clip for two reasons. First, the two children in each clip could have behaved in very different ways. Thus, if one child was bored and the other excited, the clip would be assigned both the Boredom and Excitement labels (see Table 2). Second, we recognized that a lot can happen in 30 s (the duration of the clips) resulting in changes in the internal states or social constructs which could be inferred from the children's behaviors. For example, an interaction might involve an excited child pushing the other away so they didn't have to share the touch-screen, causing the second child to sit and watch in a manner denoting boredom, this clip could be labeled with Excitement, Aggression and Bored. These labels were selected based on two considerations: (a) the events and internal states which appear available the dataset, and (b) events and internal states which would be useful to a robot which might observe or mediate such an interaction. Recognizing boredom and aimless behavior would allow a robot to appropriately encourage a child to take part in a task. Recognizing when a child is being dominant or aggressive could provide a robot with cues to mediate and balance the interaction, or request assistance from a human adult (e.g., in the case of aggressive behavior). Recognizing excitement, fun and cooperation could be used to cue positive feedback from the robot, or to signal that the robot need not interject. The selection was made independently by two of the authors, using a consensus method to reach agreement. It is important to note that interactions in this data set were minimally controlled - pairs of children from the same school class were asked to play on a touch-screen table-top for as long as they wanted. Whilst structured play options were provided, they were not enforced. The selected clips were stored on a private server for the duration of the experiment.

Similarly to the selection of clip labels, the questions were constructed by the experimenters based on the types of internal states and social constructs we might want an artificial system to recognize within a scene. The open question was a single item which asked participants “*What did you notice about the interaction?.”* The closed questions were a series of 4 unique questions concerning group dynamics, and 13 2-part questions wherein participants were asked the same question twice, once regarding the child on the left and once regarding the child on the right. Each of these 13 pairs were displayed one after the other. Otherwise, the order in which the questions were presented was random (see Appendix A for the questions and response options).

It is important to note that the ground-truth of what internal states the children were experiencing during their interactions is not available. As such, neither the labels used for clip selection and labeling, nor the inferences participants provide in their questionnaire responses can be truly validated. The labels were, therefore, also an attempt to work out what naive observers would infer from the videos.

### 2.3. Apparatus

**Figure 2:**
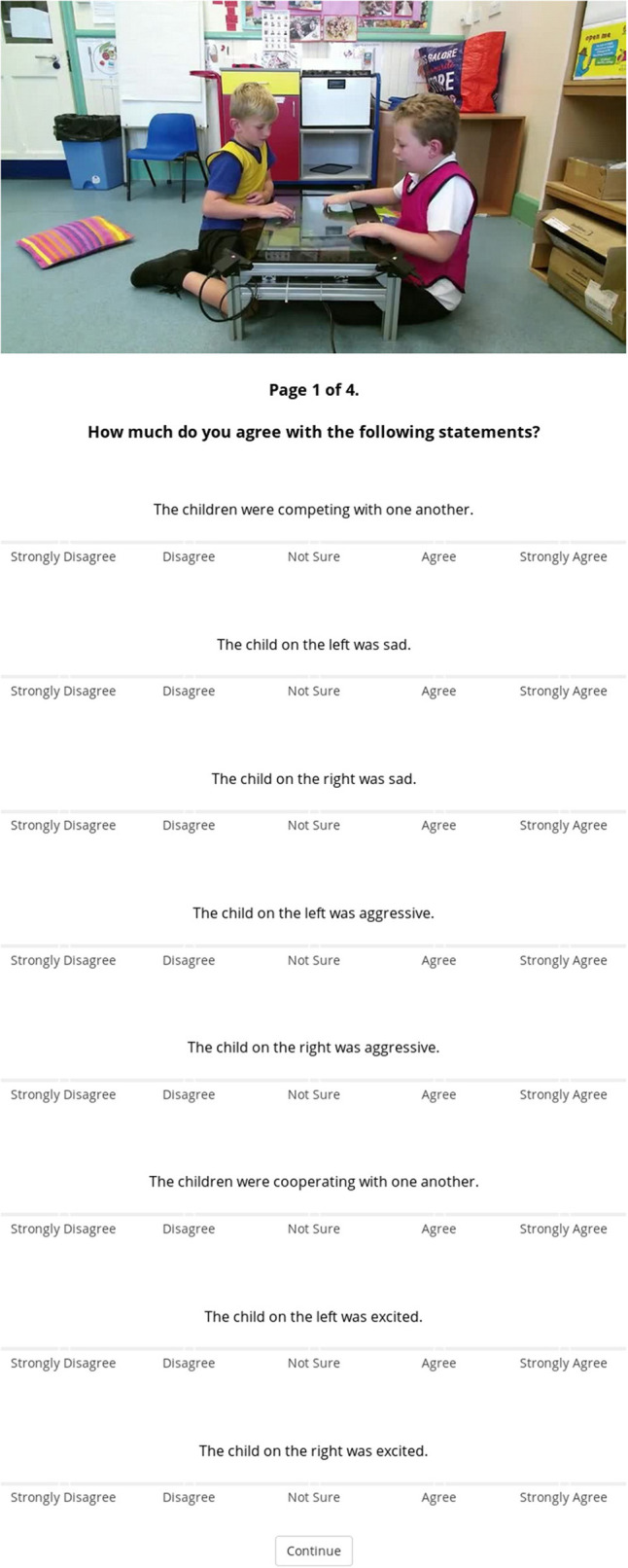
Screenshot of the online experimental setup showing the questionnaire, just after watching the video clip (here in the full-scene condition). The poster image displayed at the top is a static snapshot of the clip. Written consent for these images to be shared was obtained during collection.

The experiment was designed using the jsPsych library^3^, and remotely hosted from a private server (Figure 2 shows a screenshot of the experiment). The experiment was accessible via Amazon Mechanical Turk (MTurk) to MTurk Workers. An advert was posted on MTurk containing a link to the experiment. The remote/online nature of this study means that we had no control over the physical set-up experienced by the participants.

### 2.4. Procedure

The two video conditions were posted as separate experiments. To ensure that participants did not complete both conditions, the experiments were posted one at a time. Upon opening the experiment participants were asked to provide their MTurk ID and then shown a welcome screen. This was followed by a consent form where participants were asked to provide consent by selecting one of two response options (“I do not consent,” or “I do consent”). If participants selected “I do not consent,” the experiment would close. If they selected “I do consent” participants were able to press a “Continue” button and proceed to an instruction screen. This was followed by a series of 4 demographic questions (age, nationality, first language and gender). An instruction screen was then presented for a minimum of 3,500 ms, containing the following text:

“*During this experiment you will be shown 4 30-second clips of children interacting. The children are sat either side of a touch-screen table-top on which they can play a game. Pay particular attention to the way the children interact. After each video you will be asked some questions about what you have watched.”*

Participants could then press any button to continue on to the experimental trials.

**Figure 3:**
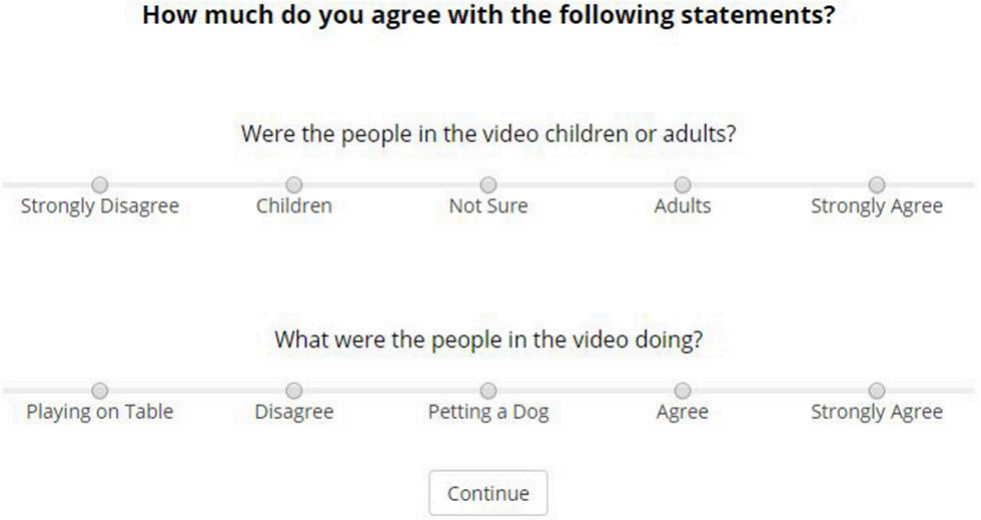
Capture of attention check questions presented at the end of the questionnaire. Single correct answer provided. Questions and responses are presented in the same format as the rest of the questions in order to test whether participants read the questions.

All participants were asked to complete 4 trials and were presented with the same series of events within each trial. Each trial started with a 30 s clip selected randomly from the list of 20, which was immediately followed by the questions. Upon completion of the fourth trial, participants were shown an additional 2 questions which acted as an attention check (see Figure 3). Responses to these questions were used to assess how attentive participants were and how diligently they completed the experiment. Participants who responded incorrectly were excluded from analysis.

Participants then viewed a debrief page which thanked them, explained the purpose of the study and attention-check questions, and provided participants with contact information if they had further questions or desired to withdraw their data. Participants were then provided with a “survey code” which was randomly generated and were instructed that they had completed the experiment and should now return to the MTurk page in order to submit their survey code. The survey codes participants submitted were later compared to those generated to validate participation and payment was authorized via the MTurk system. The experiment took between 20 and 30 min to complete.

The resulting data set is fully anonymous, and made publicly available at https://github.com/severin-lemaignan/pinsoro-kinematics-study/blob/master/fulldata.csv.

## 3. Results

All data analyses were performed with the Python pandas and sklearn toolkits. The notebook used for this article, allowing for the replication of our results, is available online, see section 5.

The responses to the open questions revealed no insights beyond those addressed in the specific questions. Therefore, the analyses of these responses are not included in this report.

### 3.1. Inter-rater Agreement

To determine inter-rater agreement and reliability, we calculated agreement scores across all 30 questions for each clip in each condition separately. This analysis was performed to examine whether participants in each condition gave similar ratings across all questions when they had viewed the same clip. High agreement would indicate that participants had interpreted similar things from a given clip, e.g., participants might all have felt that the children in a clip were being friendly and cooperative, or aggressive and competitive. Whilst this analysis does not reveal exactly what participants interpreted from the videos, it does indicate whether they gave similar ratings, and therefore reported recognizing similar states/behaviors. Given that each clip was rated by a varying subset of participants, Krippendorff's alpha (Hayes and Krippendorff, 2007) was the most appropriate metric of rater agreement (see Table 3 for number of raters and agreement per clip). The alpha scores ranged from 0.058 to 0.463 i.e., from “slight” to “moderate” agreement (Landis and Koch, 1977).

**Table 3:** Table of inter-rater agreement scores for responses to each clip in each condition.

**Clip**	**Krippendorff's Alpha (3 d.p.)**	
	**Full-Scene (N)**	**Movement Alone (N)**
1	0.446 (16)	0.186 (26)
2	0.181 (24)	0.270 (20)
3	0.393 (22)	0.369 (18)
4	0.444 (22)	0.262 (23)
5	0.328 (23)	0.283 (20)
6	0.463 (19)	0.359 (19)
7	0.091 (19)	0.236 (23)
8	0.339 (19)	0.312 (17)
9	0.097 (20)	0.058 (18)
10	0.396 (18)	0.086 (13)
11	0.280 (17)	0.234 (23)
12	0.368 (25)	0.298 (16)
13	0.334 (20)	0.189 (21)
14	0.310 (17)	0.309 (21)
15	0.422 (26)	0.242 (14)
16	0.192 (16)	0.272 (21)
17	0.273 (17)	0.183 (21)
18	0.334 (16)	0.331 (24)
19	0.415 (22)	0.304 (19)
20	0.451 (18)	0.250 (23)

A *t*-test was conducted to assess whether the two conditions differed in their agreement scores across all 20 clips. This analysis revealed that participants in the full-scene condition showed significantly higher agreement (*M* = 0.328, *SD* = 0.110) than participants in the movement-alone condition (*M* = 0.252, *SD* = 0.079) (Paired Samples *T*-Test: *t*_(39)_ = 2.95, *p* = 0.008, *d* = 0.78). These analyses show that participants viewing the full-scene clips demonstrated higher levels of agreement in their ratings than those viewing the movement-alone clips. However, participants in the latter condition still showed some agreement compared to chance (chance level Krippendorff's Alpha = 0.0; One Sample *T*-Test: *t*_(19)_ = 13.95, *p* = < 0.001, *d* = 3.12), suggesting that some internal states and social constructs were recognizable within the movement information in both conditions.

### 3.2. Automatic Labeling of Internal States

The following analysis explored the question of whether the internal states and social constructs which were available to/inferred by humans when viewing the full visual scene was also available in the movement-alone condition.

We investigated this question using supervised machine learning: would a classifier, trained to label internal states and social constructs from the full-scene ratings, then label the social situations equally well from the movement-alone ratings? If so, this would suggest that the same interaction information was recognized by, and therefore available to, participants in each video condition.

**Pre-processing** Participants' ratings were coded from 0 (*strongly disagree*) to 4 (*strongly agree*), each construct being recorded as *left*_*construct*_ and *right*_*construct*_ (see Appendix A). Before the following analyses were run, the data from the right-left paired questions was transformed so that results could be more easily interpreted in terms of what behaviors were evident in the interactions, ignoring whether it was the child on the right or the left who was exhibiting this behavior. First, for each question we calculated the absolute difference *diff*_*construct*_ = *abs*(*left*_*construct*_ − *right*_*construct*_) between the score for the left child and the right child. This score was calculated so that we could more easily see if the children were rated as behaving in the same way, or experiencing similar internal states. Examining the individual scores for each child would have meant that in order to see the dynamics between the children, each clip would have needed to be analyzed separately. Second, for each question we calculated the sum (shifted to the range [−2, 2]) *sum*_*construct*_ = *left*_*construct*_ + *right*_*construct*_−4 of the scores for both children. This score was calculated because the difference score does not contain information about the strength of the rater's belief that the behavior or internal state was evident in the clip. For example, we might have the same difference score for clips where raters believed that both children behaved aggressively and that neither child behaved aggressively. The sum score tells us the degree to which a state was identifiable in the clip.

**Multi-label classification** To test whether the same interaction information was reported in each video condition we examined whether the ratings from each condition were sufficient to identify the types of internal states or social constructs which were depicted in the videos.

The classifier was trained in a supervised manner, using the 30 ratings provided by the participants (questions from Appendix A, pre-processed as indicated above) as input, and the seven labels assigned to each clip during selection (Table 2) as the target classification classes. Because the clips could be assigned multiple labels (e.g., a given interaction can be *fun* and *cooperative* at the same time), we used a multi-label classifier (Pieters and Wiering, 2017), using 7-dimensional binary vectors (wherein a zero value denoted that a label was not present in the clip, and a value of one denoted that it was).

We compared the performances of four of classifier (random forest classifier, extra-tree classifier, multi-layer perceptron classifier and a k-Nearest Neighbor classifier, using implementations from the Python sklearn toolkit; hyper-parameters were optimized using a grid search where applicable), and eventually selected a k-Nearest Neighbor (with *k* = 3) classifier as providing the best overall classification performance.

Accuracy, precision, recall and F1 score were calculated to assess the performance of the classifier (following recommendations in Sorower (2010) and using the *weighted* implementations of the metrics available in the Python sklearn toolkit). Specifically, in the following, *Accuracy* reports the percentage of instances where the predicted labels match exactly with the actual labels; *Precision* is calculated as the ratio $\frac{tp}{tp+fp}$ of true positives divided by the total number of predicted labels (true positives + false positives); *Recall* is calculated as $\frac{tp}{tp+fn}$, i.e. the ratio true positives over the total number of labels that *should* have been found (true positives + false negatives). Finally, the *F1 score* is the harmonic average of the precision and recall, calculated as $\frac{2precision\cdot recall}{precision+recall}$.

To see how well the classifier performed, we compared performance against chance. Chance levels for these metrics were calculated by training the classifier with randomly generated labels (using the same distribution of labels as found in the real data set), and then measuring the classifier's performance on the actual testing data set.

Results are shown in Table 4. In both testing conditions, performance is poor to moderate (for instance 15.8% accuracy for the exact predictions of correct labels in the movement-alone clips), but remain markedly above chance levels (following Ojala and Garriga (2010) permutation-based *p*-value for classification significance, we found *p* = 0.02 for the full-scene classification, and *p* = 0.01 for the movement-alone classification, ruling out with high probability the null hypothesis that the classification results are due to chance).

**Table 4:** Classification results. *Full-scene* results are obtained by training the classifier on 80% of the full-scene ratings, and testing on the remaining 20%; *Movement-alone* results are obtained by training the classifier on 100% of the full-scene data, and testing on the movement-only ratings.

	**Accuracy**	**Precision**	**Recall**	**F1-measure**
Full-scene	15.1	44.5	32.0	36.1
Chance	3.7	27.3	14.0	17.4
Movement-alone	15.8	41.6	32.7	36.3
Chance	3.9	28.2	14.2	17.9

*Results are averaged over a 300-fold cross-validation. Values are given as percentages*.

Importantly, we found that prediction scores are very similar when testing the classifier on the full-scene ratings or when testing on the movement-alone ratings. This indicates that, from the perspective of automatic data classification, participants who viewed the movement-alone videos were able to report similar details as participants in the full-scene condition. This suggests that the movement-alone videos contain sufficient information to identify different internal states and social constructs.

**Figure 4:**
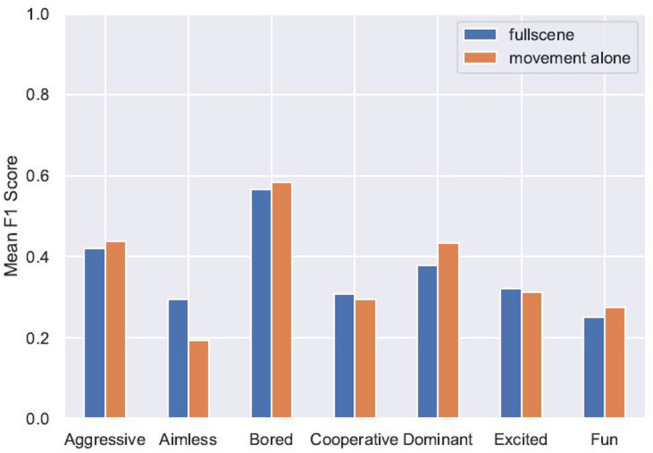
F1 scores of individual label predictions in both conditions.

To identify whether there were particular internal states or social constructs which were easier to recognize than others, the F1 score for each label was calculated. These results are reported in Table 5 and Figure 4. We can see that in both conditions the labels “Bored” and “Aggressive” have higher F1 scores than the other labels. Additionally, the F1 scores for these labels when classifying the full-scene ratings (Bored: 60.0%, Aggressive: 39.0%) are similar to the F1 scores when testing was done on the movement-alone ratings (Bored: 58.5%, Aggressive: 43.7%). This suggests that these constructs are as readily recognized when viewing the full visual scene as when viewing only body movements. In contrast, the F1 score for “Aimless” when testing on full-scene ratings is similar to the scores for most of the rest of the labels (30.3%) but drops to be much lower than any other label when testing was done on the movement-alone ratings (19.4%). This could be interpreted as showing that aimless play, whilst fairly well recognized from the ratings of full visual scene videos, is much harder to recognize from ratings produced when participants viewed only movement information.

**Table 5:** F1 scores for each independent label.

	**Aggressive**	**Aimless**	**Bored**	**Cooperative**	**Dominant**	**Excited**	**Fun**
Full-scene	42.2	29.5	56.6	30.7	37.9	32.2	25.1
Chance	18.8	17.3	11.7	18.2	20.0	18.6	11.4
Movement Alone	43.7	19.4	58.5	29.6	43.4	31.2	27.5
Chance	20.1	16.1	10.7	18.7	19.9	17.3	10.4

*See Table 4 for the meaning of each row. Values are given as percentages*.

This analysis relied on the labels assigned by some of the authors during clip selection. However, participants may have been able to recognize other internal states or social constructs not covered by these labels. In order to investigate possible latent constructs that participants in both conditions may have relied on, we next performed a factor analysis on the dataset.

### 3.3. Factor Analysis

An Exploratory Factor Analysis (EFA) was performed to explore what types of information participants reported recognizing from the videos. If similar latent constructs are found to underlie participants responses in each condition, this would support the conclusion that participants reported identifying the same types of information in each type of video. Additionally, exploring what factors load into each construct would provide an indication of what these types of information are.

**EFA** Preliminary assessments revealed a Kaiser-Meyer-Olkin (KMO) statistic of 0.89 and the Bartlett's Test of Sphericity was significant, indicating that the data was suitable for performing an EFA. EFA was performed on the ratings data from each video condition separately to examine what types of interaction information participants were able to draw from the full visual scene compared to movement information alone. We used the factor_analyzer Python module^4^ to perform the EFA, additionally using a *promax* rotation. Three factors were found to explain 44% of the variance in the full-scene ratings, and 46% in the movement-alone ratings. The factor loadings for each component can be seen in Table 6.

**Table 6:** Factor loadings for the three-factor solution using EFA, with factor loadings > 0.35.

	**Factor 1: imbalance**		**Factor 2: valence**		**Factor 3: engagement**	
	***Full-scene***	***Mov.-alone***	***Full-scene***	***Mov.-alone***	***Full-scene***	***Mov.-alone***
Diff sad	0.41	0.52				
Sum sad			0.72	0.53		0.49
Diff happy	0.49	0.53				
Sum happy				–0.51	–0.55	
Diff angry	0.40	0.62				
Sum angry			0.81	0.85		
Diff excited	0.53	0.63				
Sum excited					–0.71	
Diff calm	0.45	0.63				
Sum calm				–0.45		
Diff friendly	0.69	0.56				
Sum friendly				–0.60	–0.43	
Diff aggressive	0.78	0.79				
Sum aggressive			0.80	0.72	–0.36	
Diff engaged		0.39			0.65	0.52
Sum engaged					–0.64	–0.64
Diff distracted					0.65	0.63
Sum distracted			0.63			0.82
Diff bored		0.44			0.61	0.54
Sum bored			0.58		0.48	0.83
Diff frustrated	0.53	0.61				
Sum frustrated			0.70	0.69		
Diff dominant	0.75	0.81				
Sum dominant			0.53	0.52		
Diff submissive	0.68	0.72				
Sum submissive			0.54			

A Pearson correlation was conducted to examine the similarity of components found in the full-scene and movement-alone ratings. A strong positive correlation was found between each pair of components: for Factor 1: *r* = 0.94, *p* < 0.001; for Factor 2: *r* = 0.84, *p* < 0.001; for Factor 3: *r* = 0.81, *p* < 0.001. This supports the hypothesis that the same latent constructs are relied upon by the participants to rate social interactions, be it based on raw video footage (full-scene) or on a simplified, movement-only, stick-man-style representation (movement-alone).

By inspecting the distribution of factors loadings in Table 6, the latent constructs can be further interpreted. It appears that the first component is describing how different the children's behaviors and emotional states are, i.e. this factor describes an *imbalance* in the children,s social, behavioral, and emotional states. For instance, a high value on this scale would show that the children were reported as behaving very differently, e.g., if one child was highly engaged, the other was not very engaged at all.

The second component describes the overall *valence* of the interaction. A high value on this factor would indicate a negative, adversarial interaction where the children were rated as being sad, aggressive etc. Alternatively, a (lower) positive valence value might result from an interaction where one child was rated as being more sad or aggressive than the other child was happy. For both conditions this component has positive correlations with the *Sum* items for negative emotions and behaviors (e.g., Anger, Aggression). For the movement-alone condition, this component also has negative correlations with *Sum* items for positive emotions and behaviors (e.g., Happiness, Friendliness).

The third component is mostly describing the children's *engagement* with their task. In comparison to the other two components it contains more of a mix of *Sum* and *Difference* items, and therefore describes both how similar the children were in how engaged they were, and the overall level of engagement within the interaction. A high value on this third factor would show that the children were rated as showing different levels of engagement, but a strong indication of boredom within the interaction as a whole.

**Social Expressiveness of the EFA-Space Embedding** One may wonder whether these three factors alone would allow by themselves for an effective assessment of a social interaction, i.e. is the social “expressiveness” of our EFA factors as good as the original 26 factors? This can be investigated by re-applying the same classification methodology as used in section 3.2 to the EFA embedding of the participants' ratings.

To this end, the 26-dimensional participant ratings were projected onto the smaller, 3-dimensional, space spanned by the EFA factors (the *EFA-space*):

$$\begin{array}{l} \;M_{fullscene}^{EFA}=M_{fullscene}\cdot \Lambda_{fullscene}^{EFA}\\ M_{movementalone}^{EFA}=M_{movementalone}\cdot \Lambda_{fullscene}^{EFA} \end{array}$$

with *M*_*fullscene*_ the 396 × 26 matrix of the participants' ratings, $M_{fullscene}^{EFA}$ the 396 × 3 matrix of the participants' ratings projected onto the EFA space, and $\Lambda_{fullscene}^{EFA}$ the 26 × 3 matrix of the EFA factor loadings (Table 6). Both the full-scene clips and the movement-alone clips where projected into the same space (spanned by the factors found during the full-scene EFA).

Then, we retrained the same classifier (a kNN with *k* = 3) as in section 3.2, and tried to predict social labels from EFA-projected ratings unseen at training time. Tables 7, 8 show the results. We observe a drop of about 4–6% in performance, but still above chance.

**Table 7:** Classification results, including classification in EFA-space. *EFA-space* means that the dimensionality of the training and testing data is reduced to 3 by projecting the ratings onto the 3-dimensional space spanned by the EFA factors; non-EFA values copied from Table 4 for comparison.

	**Accuracy**	**Precision**	**Recall**	**F1-measure**
Full-scene, EFA	11.2	38.3	26.2	30.0
Full-scene	15.1	44.5	32.0	36.1
Chance	3.8	28.1	14.2	17.8
Movement-alone, EFA	11.7	35.1	27.0	30.3
Movement-alone	15.7	41.6	32.7	36.3
Chance	3.9	28.3	14.2	17.9

*Values are given as percentages*.

**Table 8:** F1 scores for each independent label, including after classification in the EFA-space.

	**Aggressive**	**Aimless**	**Bored**	**Cooperative**	**Dominant**	**Excited**	**Fun**
Fullscene, EFA	37.8	16.2	53.9	29.4	29.7	25.9	20.6
Fullscene	42.2	29.5	56.6	30.7	37.9	32.2	25.1
Chance	19.1	16.5	11.7	19.0	19.6	17.4	11.0
Movement alone, EFA	36.5	24.0	49.2	24.6	33.7	27.4	12.2
Movement alone	43.7	19.4	58.5	29.6	43.4	31.2	27.5
Chance	19.8	16.4	10.7	18.9	19.9	17.9	10.5

*Non-EFA values copied from Table 5 for comparison. Values are given as percentages*.

## 4. Discussion and Conclusion

Psychology literature has long established the importance of observing physical group behaviors to provide us with a unique window onto the agents' internal states, as well as the current state of the social interaction. Specifically, we have previous evidence of the role of *movements/actions* as an important social signal (Gallese and Goldman, 1998; Alaerts et al., 2011). The main contribution of this paper is to investigate the question of what different states are identified by observers of naturalistic interactions, looking at the (rather messy) social interactions occurring between children while playing together.

This study aimed to examine the kinds of information humans report recognizing from the movements of such naturalistic social interactions. We investigated the following question: is movement information alone (in our case, the moving skeletons of two children playing together, pictured on a uniform black background) sufficient for humans to successfully infer the internal states and social constructs experienced and present within a social interaction? Our methodology involved a between-subject, on-line study, where participants were asked to rate children's behaviors along 17 dimensions, having either watched the raw footage of short interaction videos, or only the skeletons and facial landmarks extracted from the same video clips. This resulted in about 800 unique human ratings, covering both conditions, across 20 different clips, selected for displaying a range of different internal states and social constructs.

We explored the ratings data set (which is publicly available, see the details in the following section) using two main data mining techniques. We first trained a classifier on the full-scene ratings with hand-crafted social labels to then attempt to automatically identify these social labels on the movement-alone ratings. Our results show that training our best performing classifier (a 3-kNN) on 80% of the full-scene ratings and testing on the remaining 20% results in a (cross-validated) precision of 46.2% and recall of 33.6%. We found very similar levels of precision and recall (respectively 41.6 and 32.7%) when testing on the movement-alone ratings: the assessment of the social interaction taking place between two children, made by naive observers watching a low-dimensional, movement-alone video-clip of the interaction, carries similar informational content regarding the internal states and social constructs as the original raw video footage. Based on this finding, we can tentatively conclude that whilst the movement alone videos contain fewer pieces of information, the pieces of information available are as meaningful as those in the full scene videos. Furthermore, we can assess that these pieces of information can be interpreted by human observers in a similar way as those in the full scene videos.

To better make sense of these results, we employed a second data mining technique (Exploratory Factor Analysis, EFA) to attempt to uncover underlying latent factors that would in effect embody stronger cognitive constructs, implicitly relied upon by the humans when assessing a social interaction. We ran independent EFAs on the ratings provided for the full-scene videos and those provided for the movement-alone clips.

To our surprise, the latent factors found by the EFA were strongly correlated between both conditions. In both condition, one factor was measuring the **behavioral imbalance** between the two children (i.e. how similar or dissimilar their behaviors were); a second factor reflected the **valence of the interaction**, from adversarial behaviors and negative emotions, to pro-social and positive behaviors and emotions; finally a third factor embodied **the level of engagement** of the children. These constructs may be indicative of the constructs humans use to interpret social interactions in general. Further research is needed to confirm whether or not this is the case. However, if it is it would provide further insights into how humans approach the interpretation and understanding of social interactions. That is, these three factors may represent the basic cognitive constructs humans use to understand social interactions. Consequently, HRI research could use these constructs as a basic framework for exploring human behavior for classification purposes.

Using the 3-dimensional subspace spanned by these three EFA factors, we have furthermore shown that ‘summarizing’ the internal states and social constructs inferred by the participants into the 3 latent constructs—imbalance, valence, engagement—only slightly degrades the ability of the classifier to predict the social labels associated with the interaction. This reinforces the hypothesis that these three constructs might play a foundational role in the human understanding of social interactions.

The results of both the classification analysis and EFA demonstrate that it is reasonable to expect a machine learning algorithm, and in consequence, a robot, to successfully decode and classify a range of internal states and social constructs using a low-dimensional data source (such as the movements and poses of observed individuals) as input. Specifically, whilst this study does not examine the ability to identify the correct internal states or social constructs, we have shown that, in a robust way, people agree in their reports of what they have seen both within and between conditions. As such, our study shows that, even though assessing social interactions is difficult even for humans, using skeletons and facial landmarks only does not significantly degrade the assessment. Future studies aiming to train a robotic system would ideally utilize a training dataset where the internal states and social constructs have been verified (and therefore a ground-truth is available). This study provides the evidence to guide this type of work, for example by demonstrating that training a robot to recognize aggression from movement information is likely to be more successful than recognizing aimlessness.

### 4.1. Opportunities for Future Work

Given that this work is exploratory in nature, it presents a number of opportunities for future work. First, while above chance, the accuracy of the classifier is relatively low. This may reflect the inherent difficulty of rating internal states and social constructs for an external, naive observer (such as the raters recruited for this study). The literature on emotion recognition does show that humans are able to recognize emotional states from impoverished stimuli with a high level of accuracy [e.g., 44–59% in Alaerts et al. (2011), 59–88% in Gross et al. (2012)]. Similarly, research regarding the recognition of dispositions and social behaviors indicate that computational techniques can achieve a higher recognition accuracy than the current study. For example, Okada et al. (2015) achieved around 57% accuracy in classifying dominance. However, there is some evidence to suggest that humans may not be as accurate as computational classifiers in identifying internal states as we define them here. To demonstrate, Sanghvi et al. (2011) found that whilst human observers were able to recognize engagement to an average of 56% accuracy, their best classifier achieved an 82% level of accuracy. Whilst the accuracy scores presented here are much lower, the existing literature suggests that this may be a result of the fact that humans do seem to demonstrate some difficulty in recognizing these types of states. Additionally, it is important to remember that the classifier in this study labeled the clips using the ratings of all the left/right child questionnaire items, whereas previous research has tended to use the raw visual and/or audio information for classification by both computational systems (Okada et al., 2015) and human observers (Sanghvi et al., 2011). This high dimensional input may have had the effect of diluting the specificity and causing the classifier to use irrelevant or unhelpful inputs when making classification decisions. Additionally, the low classification accuracy may result from the fact that the questionnaire used in this study might not have been good enough. As such, future research would benefit from developing and optimizing the questionnaire.

Additionally, the present study does not explore precisely which movement characteristics were useful for participants in making inferences about the internal states of the children in the videos. In this study we employed a supervised classification technique to demonstrate that social interaction assessments based on full-scene or movement-only stimuli were of similar quality–most notably, our input were ratings of social interactions by human observers. This technique is *not* practically transferable to a robot, as robots would have to directly classify the raw stimuli (a video stream or skeletons), without having access to intermediate ratings of the agents' states. Creating such a classifier is an important next step in deciphering how humans recognize internal states, and therefore in deciding how a robot or classifier can be endowed with a similar skill, for which our present results provide a solid foundation.

The fact that the internal states experienced by the children in the videos could not be validated does present a further limitation for this study. A number of datasets demonstrating one or a subset of the internal states we are interested in are available. For example, the Tower Game Dataset consists of human-human pairs collaborating on a task, and has been annotated for joint attention and entrainment behaviors reflecting cooperation and collaboration (Salter et al., 2015). Similarly, the DAiSEE dataset contains videos of individuals watching videos in an e-learning setting and is annotated for the internal states of boredom, confusion, engagement, and frustration (Gupta et al., 2016). Other datasets include: the UE-HRI annotated for engagement (Ben-Youssef et al., 2017), the ELEA annotated for perceived leadership and dominance (Sanchez-Cortes et al., 2011) among others. Replicating this experiment using a validated dataset may provide stronger classification and inter-rater agreement results. However, few ecologically-valid datasets present the range and variety of internal states as are available in the PInSoRo dataset. As such, this present research represents an important first step in framing the research methodology for analysis of complex, real-life social interactions.

### 4.2. Conclusion

The aim of this study was to identify social constructs or human internal states which a socially interactive robot could be made to recognize. Analyzing the weighted precision scores for each classification label revealed that “Aggressive” and “Bored” were classified correctly more often in both conditions, whilst “Aimless” was classified correctly much less from the movement-alone ratings. This suggests that training a robot to recognize aimlessness based on movement information might not be as successful as training recognition of boredom. Practically speaking, this finding suggests that designing a tutor robot, such as those used by L2TOR (Belpaeme et al., 2015), to recognize when a child is bored by their task based on movement information would be more successful than having the robot recognize when a child is performing the task in an “aimless” or “non-goal-directed” manner. Such a robot could then appropriately offer encouragement or an alternative task.

Additionally, these findings suggest that exploring other data sources for recognizing human internal states may reveal that certain behavioral modalities may be more useful for recognizing different states. In this way, the method we have demonstrated here can be used to streamline research aimed at teaching robots [and other classification technologies, e.g., automatic classification of security footage (Gowsikhaa et al., 2014)] to recognize human internal states. By applying this method to different types of input data, research can identify the optimal behavioral modality for recognizing a particular human internal state.

These findings have significant impact for both social psychology and artificial intelligence. For social psychology, it consolidates our understanding of implicit social communication, and confirms previous findings that humans are able to recognize socially relevant information from observed movements (Iacoboni et al., 2005; Alaerts et al., 2011; Quesque et al., 2013). For artificial intelligence, and in particular, for social robotics and human-robot interaction, it provides support for the intuition that low-dimensional (about 100 skeletal and facial points per agent vs. full video frames comprising of hundred of thousands of pixels), yet structured observations of social interactions might effectively encode complex internal states and social constructs. This provides promising support for fast and effective classification of social interactions, a critical requirement for developing socially-aware artificial agents and robots.

## 5. Resources for Replication

Following recommendations by Baxter et al. (2016), we briefly outline hereafter the details required to replicate our findings.

### 5.1. Study

The protocol and all questionnaires have been provided in the text. The code of the experiment is available at https://github.com/severin-lemaignan/pinsoro-kinematics-study/. Note that, due to data protection regulations, the children' video clips are not available publicly. However, upon signature of an ethical agreement, we can provide them to the interested researcher.

### 5.2. Data Analysis

The full recorded experimental dataset, as well as the complete data analysis script allowing for reproduction of the results and plots presented in the paper (using the Python *pandas* library) are open and available online, in the same Git repository. In particular, a iPython notebook with all the steps followed for our data analysis is available here: https://github.com/severin-lemaignan/pinsoro-kinematics-study/blob/master/analysis/analyses_notebook.ipynb.

## Ethics Statement

This study was carried out in accordance with the recommendations of Ethics guidelines of the University of Plymouth Ethics Committee, with written informed consent from all subjects. All subjects gave written informed consent in accordance with the Declaration of Helsinki. The protocol was approved by the Ethics Committee of the University of Plymouth.

## Author Contributions

MB, CE, and SL contributed to the design, running, analysis, and write-up of this study. ST and TB contributed to the supervision and funding of this study.

### Conflict of Interest Statement

The authors declare that the research was conducted in the absence of any commercial or financial relationships that could be construed as a potential conflict of interest.

## Footnotes

^1^https://freeplay-sandbox.github.io

^2^https://github.com/CMU-Perceptual-Computing-Lab/openpose/

^3^https://www.jspsych.org/

^4^https://github.com/EducationalTestingService/factor_analyzer

## A. Appendix

### A.1. Questions

Open Question: “What did you notice about the interaction?”

Specific Questions: For all of the following questions participants were asked to report how much they agreed with each statement. Answers : *Strongly Disagree / Disagree / Not Sure / Agree / Strongly Agree*

1. “The children were competing with one another.”
2. “The children were cooperating with one another.”
3. “The children were playing separately.”
4. “The children were playing together.”
6-7 “The character on the left/right was sad.”
8-9 “The character on the left/right was happy.”
10-11 “The character on the left/right was angry.”
12-13 “The character on the left/right was excited.”
14-15 “The character on the left/right was calm.”
16-17 “The character on the left/right was friendly.”
17-18 “The character on the left/right was aggressive.”
19-20 “The character on the left/right was engaged with what they were doing on the table.”
21-22 “The character on the left/right was distracted from the table.”
23-24 “The character on the left/right was bored.”
25-26 “The character on the left/right was frustrated.”
27-28 “The character on the left/right was dominant.”
29-30 “The character on the left/right was submissive.”

## References

[B1] Alaerts K. Nackaerts E. Meyns P. Swinnen S. P. Wenderoth N. (2011). Action and emotion recognition from point light displays: an investigation of gender differences. PLoS ONE 6:e20989. 10.1371/journal.pone.0020989 21695266 PMC3111458

[B2] Ansuini C. Cavallo A. Bertone C. Becchio C. (2014). The visible face of intention: why kinematics matters. Front. Psychol. 5:815. 10.3389/fpsyg.2014.00815 25104946 PMC4109428

[B3] Baxter P. Kennedy J. Senft E. Lemaignan S. Belpaeme T. (2016). From characterising three years of HRI to methodology and reporting recommendations, in The Eleventh ACM/IEEE International Conference on Human Robot Interation (Christchurch: IEEE Press), 391–398.

[B4] Becchio C. Koul A. Ansuini C. Bertone C. Cavallo A. (2017). Seeing mental states: an experimental strategy for measuring the observability of other minds. Phys. Life Rev. 24, 67–80. 10.1016/j.plrev.2017.10.002 29066076

[B5] Belpaeme T. Kennedy J. Baxter P. Vogt P. Krahmer E. E. J. Kopp S. (2015). L2TOR-second language tutoring using social robots, in Proceedings of the ICSR 2015 WONDER Workshop (Paris).

[B6] Ben-Youssef A. Clavel C. Essid S. Bilac M. Chamoux M. Lim A. (2017). Ue-hri: a new dataset for the study of user engagement in spontaneous human-robot interactions, in Proceedings of the 19th ACM International Conference on Multimodal Interaction (New York, NY: ACM), 464–472. 10.1145/3136755.3136814

[B7] Beyan C. Carissimi N. Capozzi F. Vascon S. Bustreo M. Pierro A. (2016). Detecting emergent leader in a meeting environment using nonverbal visual features only, in Proceedings of the 18th ACM International Conference on Multimodal Interaction (New York, NY: ACM), 317–324. 10.1145/2993148.2993175

[B8] Breazeal C. Gray J. Berlin M. (2009). An embodied cognition approach to mindreading skills for socially intelligent robots. Int. J. Robot. Res. 28, 656–680. 10.1177/0278364909102796

[B9] Cao Z. Simon T. Wei S. E. Sheikh Y. (2017). Realtime multi-person 2d pose estimation using part affinity fields, in CVPR (Honolulu, HI). 10.1109/TPAMI.2019.2929257 31331883

[B10] Dautenhahn K. Saunders J. (2011). New Frontiers in Human Robot Interaction, vol 2. Amsterdam: John Benjamins Publishing.

[B11] Domes G. Heinrichs M. Michel A. Berger C. Herpertz S. C. (2007). Oxytocin improves mind-reading in humans. Biol. Psychiat. 61, 731–733. 10.1016/j.biopsych.2006.07.015 17137561

[B12] Ekman P. Friesen W. V. (1971). Constants across cultures in the face and emotion. J. Personal. Soc. Psychol. 17:124. 5542557 10.1037/h0030377

[B13] Gallese V. Goldman A. (1998). Mirror neurons and the simulation theory of mind-reading. Trends Cogn. Sci. 2, 493–501. 21227300 10.1016/s1364-6613(98)01262-5

[B14] Gowsikhaa D. Abirami S. Baskaran R. (2014). Automated human behavior analysis from surveillance videos: a survey. Artif. Intell. Rev. 42, 747–765. 10.1007/s10462-012-9341-3

[B15] Gross M. M. Crane E. A. Fredrickson B. L. (2012). Effort-shape and kinematic assessment of bodily expression of emotion during gait. Hum. Move. Sci. 31, 202–221. 10.1016/j.humov.2011.05.001 21835480

[B16] Gupta A. D'Cunha A. Awasthi K. Balasubramanian V. (2016). Daisee: Towards user engagement recognition in the wild. arXiv arXiv:1609 01885.

[B17] Haidt J. Keltner D. (1999). Culture and facial expression: Open-ended methods find more expressions and a gradient of recognition. Cogn. Emot. 13, 225–266.

[B18] Han J.-H. Kim J.-H. (2010). Human-robot interaction by reading human intention based on mirror-neuron system, in 2010 IEEE International Conference on Robotics and Biomimetics (ROBIO) (Tianjin: IEEE), 561–566.

[B19] Hayes A. F. Krippendorff K. (2007). Answering the call for a standard reliability measure for coding data. Commun. Methods Meas. 1, 77–89. 10.1080/19312450709336664

[B20] Hufschmidt C. Weege B. Rder S. Pisanski K. Neave N. Fink B. (2015). Physical strength and gender identification from dance movements. Pers. Individ. Diff. 76, 13–17. 10.1016/j.paid.2014.11.045

[B21] Iacoboni M. Molnar-Szakacs I. Gallese V. Buccino G. Mazziotta J. C. (2005). Grasping the intentions of others with one's own mirror neuron system. PLoS Biol. 3, 0529–0535. 10.1371/journal.pbio.0030079 15736981 PMC1044835

[B22] Kawamura R. Toyoda Y. Niinuma K. (2019). Engagement estimation based on synchrony of head movements: application to actual e-learning scenarios, in Proceedings of the 24th International Conference on Intelligent User Interfaces: Companion (New York, NY: ACM), 25–26.

[B23] Kozlowski L. T. Cutting J. E. (1977). Recognizing the sex of a walker from a dynamic point-light display. Percept. Psychophys. 21, 575–580.

[B24] Landis J. R. Koch G. G. (1977). The measurement of observer agreement for categorical data. Biometrics 33, 159–174. 843571

[B25] Lemaignan S. Edmunds C. Senft E. Belpaeme T. (2017). The Free-play Sandbox: a Methodology for the Evaluation of Social Robotics and a Dataset of Social Interactions. arXiv arXiv:1712 02421.

[B26] Manera V. Becchio C. Cavallo A. Sartori L. Castiello U. (2011). Cooperation or competition? Discriminating between social intentions by observing prehensile movements. Exp. Brain Res. 211, 547–556. 10.1007/s00221-011-2649-4 21465414

[B27] Manera V. Schouten B. Becchio C. Bara B. G. Verfaillie K. (2010). Inferring intentions from biological motion: a stimulus set of point-light communicative interactions. Behav. Res. Methods 42, 168–178. 10.3758/BRM.42.1.168 20160297

[B28] Mather G. Murdoch L. (1994). Gender discrimination in biological motion displays based on dynamic cues. Proc. R. Soc. Lond. B 258, 273–279.

[B29] Ojala M. Garriga G. C. (2010). Permutation tests for studying classifier performance. J. Mach. Learn. Res. 11, 1833–1863. 10.1109/ICDM.2009.108

[B30] Okada S. Aran O. Gatica-Perez D. (2015). Personality trait classification via co-occurrent multiparty multimodal event discovery, in Proceedings of the 2015 ACM on International Conference on Multimodal Interaction (New York, NY: ACM), 15–22.

[B31] Okur E. Alyuz N. Aslan S. Genc U. Tanriover C. Esme A. A. (2017). Behavioral engagement detection of students in the wild, in International Conference on Artificial Intelligence in Education (Wuhan: Springer), 250–261.

[B32] Pieters M. Wiering M. (2017). Comparison of machine learning techniques for multi-label genre classification, in Benelux Conference on Artificial Intelligence (Groningen: Springer), 131–144.

[B33] Pollick F. E. Paterson H. M. Bruderlin A. Sanford A. J. (2001). Perceiving affect from arm movement. Cognition 82, B51–B61. 10.1016/S0010-0277(01)00147-0 11716834

[B34] Quesque F. Lewkowicz D. Delevoye-Turrell Y. N. Coello Y. (2013). Effects of social intention on movement kinematics in cooperative actions. Front. Neurorobot. 7:14. 10.3389/fnbot.2013.00014 24109450 PMC3790102

[B35] Salter D. A. Tamrakar A. Siddiquie B. Amer M. R. Divakaran A. Lande B. Mehri D. (2015). The tower game dataset: a multimodal dataset for analyzing social interaction predicates, in 2015 International Conference on Affective Computing and Intelligent Interaction (ACII) (Xi'an: IEEE), 656–662.

[B36] Sanchez-Cortes D. Aran O. Mast M. S. Gatica-Perez D. (2011). A nonverbal behavior approach to identify emergent leaders in small groups. IEEE Trans. Multi. 14, 816–832. 10.1109/TMM.2011.2181941

[B37] Sanghvi J. Castellano G. Leite I. Pereira A. McOwan P. W. Paiva A. (2011). Automatic analysis of affective postures and body motion to detect engagement with a game companion, in Proceedings of the 6th international conference on Human-robot interaction (New York, NY: ACM), 305–312.

[B38] Schrempf O. C. Hanebeck U. D. (2005). A generic model for estimating user intentions in human-robot cooperation, in ICINCO (Barcelona), 251–256.

[B39] Shaker N. Shaker M. (2014). Towards understanding the nonverbal signatures of engagement in super mario bros, in International Conference on User Modeling, Adaptation, and Personalization (Aalborg: Springer), 423–434.

[B40] Sorower M. S. (2010). A Literature Survey on Algorithms for Multi-Label Learning. Corvallis: Oregon State University.

[B41] Tracy J. L. Robins R. W. (2008). The nonverbal expression of pride: evidence for cross-cultural recognition. J. Personal. Soc. Psychol. 94:516. 10.1037/0022-3514.94.3.516 18284295

[B42] Vernon D. Thill S. Ziemke T. (2016). The role of intention in cognitive robotics, in Toward Robotic Socially Believable Behaving Systems-Volume I (Springer), 15–27.

[B43] Walker-Andrews A. S. (1997). Infants' perception of expressive behaviors: differentiation of multimodal information. Psychol. Bull. 121:437. 9136644 10.1037/0033-2909.121.3.437

